# Preview of the 2026 ABRF Annual Meeting

**Published:** 2026-03-06

**Authors:** Deborah Hollingshead, Kathleen Brundage

**Affiliations:** 1 School of Pharmacy University of Pittsburgh; 2 Flow Cytometry & Single Cell Core Facility West Virginia University

**Keywords:** ABRF, core laboratory

## Abstract

The 2026 ABRF annual meeting, scheduled to take place in Pittsburgh from March 28 to 31, is anticipated to be a highly rewarding, vibrant, and engaging event for core laboratory personnel at all levels and across all technological disciplines.

The 2026 ABRF Annual Meeting, happening in Pittsburgh from March 28–31, promises to be professionally rewarding, dynamic, and engaging for core lab personnel of all levels and technological specialties. A little taste of what to expect at the meeting can be found in the following sections.

## Opening Keynote Speaker

Mark Christiansen, head of UCL Genomics at the University College of London, is our opening keynote speaker. Mark will share his story of growing UCL Genomics from a small institutional core lab to the center of a global network of research collaborations on a cost recovery basis. Anyone who met Mark at the 2025 meeting in Las Vegas will confirm that this will be a high-energy entertaining talk with great professional value.

## Awards

The recipient of the ABRF Award for 2026 is Dr. John R. Yates, PhD, Ernest W. Hahn Professor, Departments of Molecular Medicine and Neurobiology, Scripps Research Institute. Dr. Yates’s work within proteomics has transformed the field through development of the SEQUEST algorithm for protein identification and methods to study complex protein mixtures. His talk promises to be informative and inspirational.

Mariana De Niz, Nikon Imaging Center Manager at Northwestern University, will receive the ABRF Award for Diversity, Equity, and Inclusion (DEI). The ABRF DEI Award is given to individuals or groups who have made significant contributions toward creating a more equitable and diverse scientific community. Through her work as an active advocate and mentor for scientists from underrepresented backgrounds, working with students from low resource areas of Mexico, and translating bioimaging educational materials to reduce language barriers, Dr. De Niz has been a leader in creating an equitable scientific community.

## New This Year - Mini-workshops

Following the success of the AI and Bioimage analysis mini-workshop offered in 2025, this year’s program will include four opportunities to dive more deeply into a topic with a small group in an extended double time slot. Mini-workshop offerings include a repeat of AI and Bioimaging as well as Marketing Your Core, 3D Printing and Power Apps. Attendance is limited so preregistration (available within the general meeting registration) is required. A small fee is being charged.

## Plenary and Concurrent Sessions

Plenary session topics include a presentation from Research!America (the advocacy alliance that ABRF has recently joined), a talk led by Kelly Vere (a leader in the “more than just a technician” movement in the UK), and discussions focused on information about changing regulations around animal use in research, the latest genomic technologies, and more. Concurrent session topics include technical presentations in imaging, cytometry, genomics, proteomics, explorations of core operations, administration, and the unique challenges of nonbiologically oriented cores as well as several sessions featuring updates from ABRF research groups. Lightning talks are back this year as are posters. Learn more about the exciting science being done by your colleagues in other cores across the country.

## Exhibition Hall

We all know that this meeting would not be possible without the support ABRF gets from our sponsors. Make sure to visit the vendor booths to see their latest innovations and instrumentation. Attending one or more of the tech showcases is a great opportunity to take a deeper dive into the latest offerings from some of the meeting sponsors. As in years past, don’t forget to stop by the lightning talks and posters. Learn more about the exciting science being done by your colleagues in other cores across the country.

## Wellness

Back by popular demand, the therapy dogs will be around during the meeting to give you a chance to love on some very affectionate pups. For those looking for a way to unwind, relax, or decompress, there will be morning yoga and the walking group.

## Closing Keynote Speaker

Pursuing our theme of Innovating at the Intersection of Science, Technology, and Collaboration, sessions on communicating science, sharing our stories, breaking silos, and nonbiological cores will be followed by closing keynote speaker Bryan Welm. Bryan is a sculptor who also happens to be an assistant professor of surgery in the Huntsman Cancer Center, University of Utah. He will tell us about the creative process for his work “celebrating the beauty of science and the biological world.”

## Closing Reception

Don’t forget to join us for the closing reception at Sienna Mercato, a multilevel Italian restaurant with a glass roof (retractable) rooftop patio. It is just a short walk from the convention center and meeting hotel. It is a great chance to have a final meet up with old and new friends/colleagues before we all scatter to our home bases.

## About the Meeting Location

Our venue is the David Lawrence Convention Center in downtown Pittsburgh, PA, in the heart of the city’s cultural district and within walking distance of the Strip, a pedestrian friendly neighborhood featuring a variety of opportunities for shopping, dining, and entertainment. Nearby attractions include the Andy Warhol Museum, the August Wilson African American Cultural Center, several theaters offering live performances, and the Fort Pitt Museum.

Hope to see you in “the Burgh!”

